# Dry fractionation of chickpea flour: balancing protein enrichment, coproduct functionality and environmental sustainability

**DOI:** 10.3389/fnut.2026.1984022

**Published:** 2026-10-08

**Authors:** Nicola Cesario Angelino, Tryfon Kekes, Fotini Drosou, Jürgen Zeller, Milena Corredig

**Affiliations:** 1 Department of Food Science, Aarhus University, Aarhus, Denmark; 2 Laboratory of Process Analysis and Design, School of Chemical Engineering, National Technical University of Athens, Athens, Greece; 3 Hosokawa Alpine AG, Augsburg, Germany

**Keywords:** chickpea, dry fractionation, Life Cycle Assessment, protein, starch, sustainability

## Abstract

Dry fractionation is a resource-efficient approach to produce protein-rich fractions from legumes. However, this process has mainly been optimized for protein content and yield of the fine fraction, while the functional properties of the coarse fraction have received less attention. Moreover, its environmental impact has generally been assessed in separate studies. To date, these aspects (fine fraction composition and yield, coarse fraction functionality and environmental impact) have not been considered together to optimize the dry fractionation process. This study evaluated dry fractionation of chickpea flour, performed with industrial scale equipment, under four operating conditions. The main goal was to determine to what extent a higher degree of refinement is justified when fraction composition, coarse fraction functionality and life cycle environmental impact are considered simultaneously. A clear purity-extraction efficiency trade-off was observed across all conditions: the least selective configuration achieved the highest protein extraction efficiency (59%) and fine fraction yield (36.19%) with moderate protein enrichment (36.5 g/100 g DM), while the most selective configuration reached protein content of up to 46.1 g/100 g DM but at a high cost in yield. The limited separation selectivity can be attributed, at least partly, to the overlap in particle size between starch granules and protein-rich particles in chickpea flour. The coarse fractions also differed in composition, which determined their functional properties. The coarse fraction obtained under the least selective conditions exhibited the highest extent of starch granule swelling, disruption and retrogradation during hydrothermal treatment, and formed the stiffest gel network, consistent with its elevated starch content. Life cycle assessment revealed that environmental impacts increased markedly with fractionation intensity when the whole burden was attributed to the fine fraction, decreased under mass allocation, and increased only moderately under economic allocation. This showed that the environmental performance strongly depends on how the coarse fraction is valorised. These findings highlight the three-way trade-off between protein purity, coarse fraction functional properties, and environmental sustainability, and demonstrate that coarse fraction valorisation is central to the overall viability of dry fractionation as a sustainable food ingredient production strategy.

## Highlights

- First integrated assessment of industrial scale chickpea dry fractionation, including coarse fraction functionality and environmental impact.
- Functional properties of coarse fraction change significantly with classifier configuration.
- The environmental cost of higher protein purity depends on how the coarse fraction is allocated.
- Coarse fraction composition influences starch functionality, gelation and pasting properties.

## 1 Introduction

In recent years, dry fractionation has emerged as a more sustainable protein concentration process than the most commonly used aqueous extractions (1). Dry fractionation exploits the differences in particle size, shape, and density between protein-rich and starch-rich particles in legume flours, enabling their separation through air classification systems combined with controlled airflows and size-selective devices (2). No water or energy-intensive steps are present in the process, making it less resource demanding than methods based on solubilization of the legume flours and drying of the protein fraction through spray-drying (3). To date, optimization of dry fractionation has largely focused on the trade-off between protein concentration and yield of the fine fraction (2, 4, 5). However, a more holistic approach is needed, whereby the balance between process optimization, environmental footprint and ingredient functionality needs to be addressed. Although some studies have characterized the functional properties of the fine fraction (6, 7), the coarse fraction has received much less attention. This is a significant gap, since the coarse fraction constitutes the main process output in terms of volume, and its valorisation is therefore central to the overall economic and environmental sustainability of the process.

Dry fractionation presents two limitations: first, the obtainable protein content in the fine fraction (around 60–70%) (8) is substantially lower than that of isolates produced by aqueous extractions and isolations (75–90%) (9, 10). Second, a large volume of side stream, the coarse fraction, is produced. Its composition and volume vary depending on process conditions. To overcome the limitations of dry fractionation, hybrid approaches combining it with aqueous extractions and fractionation steps have been proposed (11). Chickpea (*Cicer arietinum L.*) is among the legumes of interest, given its widespread cultivation, nutritional profile, and potential as a value-added protein source (12). While much work has been published on the fractionation of pea and faba bean (1, 4, 6, 7, 11) less data are available for chickpea flour dry fractionation, such as the influence of raw material composition, milling conditions, and process parameters (2, 13). A recent report (14) has demonstrated that dry extracted chickpea concentrate could be obtained with about 44% protein, with a techno-functional performance superior to that of the corresponding flour; however, no Life Cycle Assessment (LCA) data has been reported.

The aim of this study was to evaluate the potential of dry fractionation to produce chickpea protein concentrate through a more comprehensive approach than conventional yield- and composition-based assessments. LCA was performed using multiple functional units. Furthermore, attributing the full burden to the fine fraction was compared to applying mass and economic allocation between the fine and coarse fraction. A study with industrial scale equipment was performed using the same raw material and initial milling process. This ensured full comparability between samples, and an appropriate assessment of the effect of different refining levels on yields, starch fraction functionality and environmental footprint. Process parameters were examined not only for their effect on separation performance, but more importantly also for their impact on the functionality of the co-product (the starch-rich coarse fraction), providing a unique opportunity to test the effect of starch purity on the functionality of this side stream.

## 2 Materials and methods

### 2.1 Materials

700 kg of chickpeas were kindly supplied by Molino Peila SpA (Turin, Italy) and were dehulled by the supplier.

### 2.2 Dry fractionation process

Dry fractionation was carried out at the industrial scale facility of Hosokawa Alpine AG (Augsburg, Germany). The processing line consisted of a Zirkoplex classifier mill (ZPS 200) coupled with an Alpine Turboplex air classifier (ATP 200). A preliminary milling trial was conducted at constant mill speed (5,550 rpm) to evaluate the effect of different mill classifier wheel speeds (2,200 and 2,400 rpm) on powder particle size distribution and processability. Based on these tests, the fraction milled at 2200 rpm was selected for the dry fractionation trials. Since no relevant difference in particle size distribution was observed between the milling speeds (average particle size ≈ 15.6 µm), the lowest speed (2200 rpm) was chosen, as it was assumed to be the least energy-intensive. Different operating conditions, and therefore different cut points, were applied, yielding fractions that differed in protein enrichment. Table 1 summarizes the experimental treatments applied. Two types of classifier wheels were used. The wide wheel has straight, single lamellae. This model differs from the standard wheel in the larger spacing between the lamellae. The NG (New Generation) wheel has an additional lamella welded onto each blade. This geometry changes the flow pattern inside the wheel, applying a solid-body vortex instead of the conventional potential vortex. It therefore results in a different cut point at the same rotational speed. The NG wheel is also designed to reduce aerodynamic resistance and pressure drop, lowering fan power requirements. The two wheels were interchanged within the same air classifier (ATP 200), with all other components unchanged. For the wide wheel, a single classifier wheel speed (6,000 rpm) was used, while for the NG wheel three speeds (4,500, 5,500 and 6,500 rpm) were tested to determine the effect of speed on the composition and functionality of the fractions.

**Table 1:** Process parameters and output fractions obtained in the four dry fractionation trials of chickpea flour.

Trial number	Process parameters		Products	
	Classifier wheel	Classifier speed (rpm)	Fine fraction	Coarse fraction
1	Wide	6,000	W60F	W60C
2	NG	4,500	NG45F	NG45C
3	NG	5,500	NG55F	NG55C
4	NG	6,500	NG65F	NG65C

Since the trials were conducted in batch mode rather than a continuous industrial operation, the fine fraction yield was estimated from the mass balance of the protein rather than by direct gravimetric measurements:


$$Y_{f}=\frac{P_{i}-P_{c}}{P_{f}-P_{c}}$$

where *Y_f_* is the yield (mass fraction) of the protein-rich fine fraction, *P_i_*, *P*_*f*_ and *P_c_* the protein concentration (g/100 g) in the original flour, the protein-rich fine fraction and the starch-rich coarse fraction.

The protein extraction efficiency ( $E_{\text{prot}}$ ) was calculated as the proportion of total feed protein recovered in the fine fraction (15):


$$E_{\text{prot}}=\frac{Y_{f}\times P_{f}}{P_{i}}\times 100$$

where $Y_{f}\;$ is the mass-based yield of the fine fraction (expressed as a decimal), and $P_{f}\;$ and $P_{i}\;$ are the protein contents (g/100 g dry matter) of the fine fraction and the original flour feed, respectively.

The yields were calculated by assuming that the mass balance closes completely between the fine and coarse fractions (i.e., $Y_{f}\;$ + $Y_{c}\;$ = 1), with no material losses during fractionation. This assumption is based on the intended application of the process in a continuous industrial fractionation line, where material losses are expected to be negligible relative to the total feed mass.

### 2.3 Fine and coarse fraction characterization

#### 2.3.1 Compositional analysis

The concentration of protein present in the various fractions was determined using the Dumas combustion method, applying a nitrogen-to-protein conversion factor of 5.7. Lipid content was quantified gravimetrically following chloroform/methanol extraction. Briefly, 100 mg of sample was hydrated in 600 μL of 0.01 M phosphate buffer (pH 7.0) and stirred overnight at 4 °C. A 4 mL aliquot of chloroform/methanol (2:1, v/v) was added and stirred for 1 h, after which samples were centrifuged at 1,500 g for 10 min at 10 °C. The supernatant was collected and the pellet re-extracted with 3 mL of the same solvent mixture. Combined supernatants were washed with 3.5 mL of 0.73% (w/v) NaCl solution and centrifuged again under the same conditions. The lower organic phase was transferred into pre-weighed glass tubes and the solvent evaporated under vacuum at temperatures not exceeding 40 °C, after which lipid content was determined gravimetrically. Moisture content was measured with a halogen moisture analyzer (HR73, Mettler Toledo, Switzerland) by drying approximately 350 mg of sample at 105 °C to constant weight. Ash content was subsequently determined by incineration of 500 mg of sample in a muffle furnace at 550 °C for 5 h. Total carbohydrates were estimated by difference on a dry basis. Starch content was quantified using a commercial enzymatic kit (Total Starch Assay Kit AA/AMG, Megazyme, Ireland).

#### 2.3.2 Size distribution of the powder fractions

The particle volume distribution of the chickpea sample was determined at Hosokawa Alpine laboratory facilities, using a light scattering HELOS (H3509) system equipped with an OASISDRY disperser (R4 lens, measurement range 0.5–350 μm; Sympatec, Clausthal-Zellerfeld, Germany). The powder was fed by a VIBRI dosing unit set to a flow rate of 60% and a gap width of 2 mm. Dispersion was achieved with compressed air at 2 bar and a negative pressure of 99 mbar. The measurement was started automatically at an optical concentration ≥ 2.1% and stopped after at least 5 s once the concentration was ≤ 1.9% or after a maximum measuring time of 99 s. Laser obscuration during measurement was approximately 6.9%. A single measurement was performed by the Hosokawa Alpine for each sample. Data were evaluated with PAQXOS 5.0 software using the Fraunhofer scattering model. Characteristic diameters D_90_, as well as the volume-weighted mean diameter D[4,3], were extracted from the density volume distributions.

#### 2.3.3 Scanning electron microscopy

Powder morphology was investigated by field emission gun scanning electron microscopy (FEG-SEM) using a TESCAN CLARA instrument (model S8151). Images were collected with a secondary electron (SE) detector at an accelerating voltage of 5 kV, a magnification of ~3200x and a working distance ranging from 8 to 11 mm, adjusted according to sample surface topography. Samples were mounted on aluminum stubs covered with carbon adhesive tabs and sputter-coated with ~6 nm of palladium prior to observation to reduce surface charging. Two images were acquired per sample to confirm structural consistency. Field of view and magnification were slightly adjusted as needed to optimize focus and highlight microstructural features of interest. Astigmatism, brightness, and contrast were individually optimized for each field. Starch granule size was estimated from SEM micrographs using Fiji/ImageJ v2.14.0 (National Institutes of Health, Bethesda, MD, United States). Individual granules were manually outlined by fitting a circle to the widest particle dimension, corresponding to the maximum Feret’s diameter. A minimum of 100 granules per sample were measured to ensure a representative size distribution, and results are expressed as mean ± standard deviation.

#### 2.3.4 Pasting and gelling properties of coarse fractions

Pasting properties of the coarse fractions were determined using a Rapid Visco Analyzer (RVA 4800, Perten Instruments, Sweden) following AACC Approved Method 22–02.01 (16). All samples were prepared at a concentration of 10% (w/w) on dry matter basis. Samples were loaded into the RVA canister at room temperature, equilibrated to 50 °C, and subsequently heated to 95 °C at a rate of 12.5 °C/min. This temperature was maintained for 5 min before cooling back at 50 °C at the same rate. Stirring was applied at 960 rpm during the first minute and reduced to 160 rpm for the time of the analysis. The parameters used for the analysis were defined as in literature (17, 18). After the hydrothermal treatment and overnight cooling at 4 °C, the resulting gels were then further analyzed regarding their microstructure and viscoelastic properties.

The microstructure of the gels formed by the coarse fraction was evaluated using confocal laser scanning microscopy. Proteins were stained using Rhodamine B, and fluorescein isothiocyanate (FITC) was also used to distinguish starch granules (19, 20). Each staining solution was prepared at 0.1 mg/mL, with FITC dissolved in acetone and Rhodamine B dissolved in water. Aliquots (10 μL) of each solution were deposited onto concave microscope slides and acetone was allowed to evaporate prior to sample application. Approximately 0.1 g of gel was then placed on the slide, covered with a 24 × 24 mm coverslip, and sealed at the edges with nail polish. Dyes were left to diffuse overnight before imaging. Confocal laser scanning microscopy was performed using a Nikon Eclipse TI2 Racon instrument (Minato, Tokyo, Japan) with a 20 × objective and an image resolution of 2048 × 2048 pixels. Excitation wavelengths of 488 nm and 561 nm were applied using Ar/Kr and HeNe lasers, respectively.

The viscoelastic properties of the gels obtained using the RVA were characterized at 20 °C using a rheometer (HR10, TA Instruments, USA) fitted with a 20 mm parallel cross-hatched plate geometry. For this analysis, the gels were cast into cylindrical molds of 20 mm diameter and 3 mm height and then cooled overnight. A fixed gap of 2.7 mm was set, corresponding to 10% less than the sample thickness, to ensure adequate contact between the sample and the plates. An equilibration time of 3 min was applied prior to measurement. Samples were first subjected to a strain sweep (0.1–100% strain, 1 Hz) to identify the linear viscoelastic region (LVE). A frequency sweep (0.1–100 rad/s) was subsequently performed at 1% strain on a second gel from each batch. Storage modulus (G’) and loss modulus (G”) were recorded throughout both tests.

### 2.4 Life cycle assessment

Life Cycle Assessment (LCA) was performed in accordance with the ISO 14040:2006 and ISO 14044:2006 recommendation series (21), using the LCA for Experts software (version 10.6.2.9, Sphera Solutions GmbH, Stuttgart, Germany), with ReCiPe 2016 Hierarchist being selected as the impact assessment methodology for the analysis. The primary goal of the LCA analysis was to evaluate the environmental performance of dry fractionation, and two different functional units (1 kg of produced fine fraction and 1 kg of protein contained in the fine fraction) were selected for the present work to perform a detailed assessment of the environmental impacts of dry fractionation. Additionally, two allocation scenarios were considered for the co-products (fine and coarse fractions), namely mass and economic allocation. Mass allocation was based on the obtained masses of the two fractions:


$$\text{Mass Allocation facto}r_{\text{fine}}=\frac{m_{\text{fine}}}{m_{\text{fine}}+m_{\text{coarse}}\;}$$

$$\text{Mass Allocation facto}r_{\text{coarse}}=1-\text{Mass Allocation facto}r_{\text{fine}}$$

where *m_fine_* and *m_coarse_* are the obtained masses of the fine and coarse fractions, respectively. Economic allocation was based on the relative economic value of the two fractions (2.00 €/kg and 0.80 €/kg for the fine and coarse fraction, respectively) and calculated from their respective mass yields and unit prices:


$$\text{Economic Allocation facto}r_{\text{fine}}=\frac{Y_{\text{fine}}*E_{\text{fine}}}{(Y_{\text{fine}}*E_{\text{fine}})+(Y_{\text{coarse}}*E_{\text{coarse})}}$$

$$\begin{array}{l} \text{Economic Allocation facto}r_{\text{coarse}}\\ =1-\text{Economic Allocation facto}r_{\text{fine}} \end{array}$$

where $Y_{\text{fine}}\;$ and $Y_{\text{coarse}}\;$ are the mass-based yield of the fine and coarse fractions (expressed as a decimal), and $E_{\text{fine}}\;$ and $E_{\text{coarse}}\;$ are the economic values of the fine and coarse fractions, respectively.

The system boundaries were defined as follows: As the scope of the present study was to assess the environmental performance of dry fractionation in producing protein-rich flours, a “cradle-to-gate” approach was selected, focusing on the processes involved until the acquisition of the protein-rich flour. The involved processes include all activities related to the cultivation of chickpeas and the subsequent steps of milling and dry fractionation. For the LCA analysis, data were collected using reliable databases and following communication with relevant industry. Specifically, the data related to the cultivation of chickpeas modeled using the latest available Ecoinvent database (version 3.12), while data related to the milling and dry fractionation were obtained by Hosokawa Alpine (Augsburg, Germany). Electricity consumption was modeled using the EU electricity grid mix from the Sphera Managed LCA Content (MLC) database, 2026.2 edition, implemented in LCA for Experts. The EU electricity mix was used consistently for all fractionation scenarios. The electricity consumption of the milling and air classification steps was calculated from the measured power demand and the operating time of each trial:


E=P×t

where E is the electricity consumption (kWh), P the measured electrical power demand (kW) and t the operating time (h). Consumption was then expressed per unit mass of processed material and converted to MJ for inclusion in the Life Cycle Inventory (Table 2).

**Table 2:** Life cycle inventory (LCI) of the different dry fractionation trials.

Process	Flow	In/Out	Value	Unit
Milling	Chickpeas	In	100	kg
	Electricity	In	32.4	MJ
	Chickpea flour	Out	100	kg
W60F dry fractionation	Chickpea flour	In	100	kg
	Electricity	In	17.97	MJ
	Fine fraction	Out	36.19	kg
	Coarse fraction	Out	63.81	kg
NG45F dry fractionation	Chickpea flour	In	100	kg
	Electricity	In	17.99	MJ
	Fine fraction	Out	24.87	kg
	Coarse fraction	Out	75.13	kg
NG55F dry fractionation	Chickpea flour	In	100	kg
	Electricity	In	17.98	MJ
	Fine fraction	Out	16.90	kg
	Coarse fraction	Out	83.10	kg
NG65F dry fractionation	Chickpea flour	In	100	kg
	Electricity	In	18.03	MJ
	Fine fraction	Out	7.70	kg
	Coarse fraction	Out	92.30	kg

### 2.5 Statistical analysis

A one-way ANOVA was conducted for the compositional analysis and RVA measurements, each performed in triplicate. A Tukey test was applied as post-hoc test to identify differences between specific group means, with a significance level at *α* = 0.05. Statistical analysis was performed using RStudio (version 2025.05.01 + 513).

## 3 Results and discussion

### 3.1 Composition of the fractions

The composition of the various fine fractions obtained during dry fractionation is reported in Table 3. As shown by the relationship between protein content and protein extraction efficiency (Figure 1), there was a clear trade-off in the separation process. The wide wheel classifier, run at 6,000 rpm (W60F), resulted in the highest protein extraction efficiency (59%), but with a limited (1.6-fold) enrichment in protein, relative to the feed flour (from 22 to 36 g/100 g DM). At this setting, the lower cutting point results in a broader range of particles to be carried into the fine fraction alongside protein bodies, and this includes more starch granules, maximizing the recovery at the expense of purity (5). Using the NG-wheel classifier at different rotational speeds progressively improved the protein purity in the fine fraction, reaching 46 g/100 g DM at 6500 rpm (NG65F), corresponding to a 2-fold increase in the protein content. However, this increase in the NG65F process was at the expense of a substantial reduction in protein extraction efficiency (16%) and fine fraction yield (8%), in other words, much more protein was now present in the co-product, the coarse fraction (NG65C), using these conditions. In conclusion, the rotational speed (tested in the NG-wheel configuration) was shown to be critical to reaching high protein content, as previously reported (2, 5); however, under the tested conditions, running at rotational speed > 5,500 rpm the enrichment reached a plateau, while severely reducing process efficiency. Since energy input was comparable across trials (Table 2), such a reduction in yield is expected to increase the environmental burden per unit of product. The marked reduction in starch content in the fine fraction with increasing rotational speed, together with the related changes in total carbohydrates, also suggests a relative accumulation of non-starch carbohydrates, potentially including fiber, in the fine fraction.

**Table 3:** Proximate composition of chickpea flour, fine (W60F, NG45F, NG55F, NG65F) and coarse (W60C, NG45C, NG55C, NG65C) fractions obtained during the four dry fractionation trials.

Sample	Protein (g/100 g dm)	Lipid (g/100 g dm)	Carbohydrates (g/100 g dm)	Ash (g/100 g dm)	Moisture (%)	Starch (g/100 g dm)
Flour	22.2 ± 0.5^c^	9.3 ± 0.7^d^	64.2 ± 1.1^d^	4.3 ± 0.1^e^	8.04 ± 0.26ᵈ	36.1 ± 1.2^c^
W60F	36.5 ± 1.5^b^	11.6 ± 0.7^c^	45.7 ± 1.8^e^	6.2 ± 0.0^d^	6.04 ± 0.41ᵃᵇ	15.8 ± 0.6^d^
NG45F	39.6 ± 0.0^b^	11.3 ± 0.4^c^	42.4 ± 0.4^e^	6.7 ± 0.0^c^	6.01 ± 0.51ᵃᵇ	7.4 ± 0.2^e^
NG55F	45.0 ± 0.8^a^	13.1 ± 0.3^b^	34.9 ± 0.4^f^	7.0 ± 0.0^b^	6.03 ± 0.20ᵃᵇ	0.4 ± 0.2^f^
NG65F	46.1 ± 0.6^a^	14.9 ± 0.5^a^	31.6 ± 0.4^f^	7.4 ± 0.1^a^	5.53 ± 0.28ᵇ	0.2 ± 0.1^f^
W60C	14.1 ± 0.0^e^	6.6 ± 0.7^e^	76.3 ± 0.7^a^	3.0 ± 0.0^h^	7.94 ± 0.13ᶜᵈ	47.6 ± 1.9^a^
NG45C	16.5 ± 0.2^e^	6.6 ± 0.4^e^	73.5 ± 0.3^ab^	3.4 ± 0.0^g^	7.59 ± 0.50ᶜᵈ	38.8 ± 0.7^bc^
NG55C	17.6 ± 0.1^de^	6.9 ± 0.1^e^	72.2 ± 0.1^bc^	3.3 ± 0.0^g^	6.68 ± 0.94ᵃᵇᶜ	41.3 ± 2.9^b^
NG65C	20.2 ± 3.0^cd^	7.2 ± 0.3^e^	69.0 ± 3.2^c^	3.6 ± 0.0^f^	6.97 ± 0.39ᵃᶜᵈ	42.9 ± 3.7^ab^

Values are expressed as mean ± standard deviation. Different superscript letters within each column indicate significant differences (*p* < 0.05). dm, dry matter.

**Figure 1:**
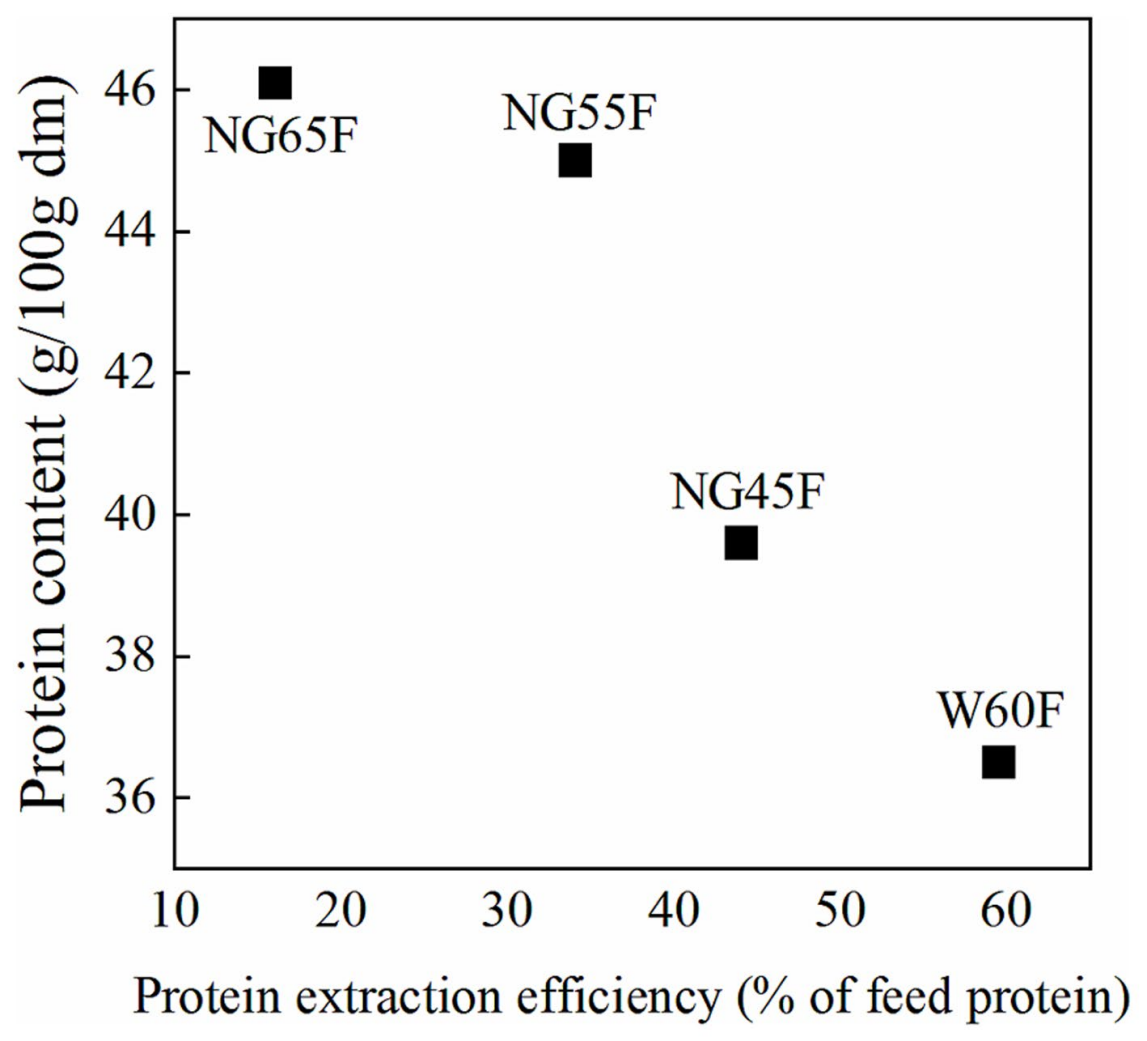
Dry fractionation performance of chickpea flour across classifier configurations. Fine fraction protein content versus protein extraction efficiency relative to the feed is shown. NG65F, NG55F and NG45F: fine fractions obtained with the new-generation classifier wheel at 6500, 5500 and 4500 rpm, respectively; W60F: fine fraction obtained with the wide classifier wheel at 6000 rpm.

The coarse fraction, which accounted for approximately 64% of the total output with the wide wheel classifier and from 75 to 92% with the NG-wheel configurations, underwent significant compositional changes depending on the process conditions. This provided a great opportunity to evaluate the effect of composition on the functionality of the coarse fraction derived from the same initial raw material, with the same milling conditions. The W60C showed the highest level of starch and lowest level of protein, and the results shown in Table 3 suggest that with the NG-wheel conditions, a substantial proportion of protein-rich particles was not selectively captured in the fine fraction but instead redistributed to the coarse stream. This redistribution of protein, together with the associated decrease in starch content, may affect the functional properties of the coarse fraction. The limits in separation efficiency for chickpea compared to other legumes have been previously attributed to the small starch granule size, low particle density, and elevated fat content of chickpeas, all of which may reduce air classification performance (2).

### 3.2 Microstructure and particle size distribution

Figure 2 compares representative images of the initial chickpea flour, and the fine and coarse fractions obtained after dry fractionation. Their particle size distribution is summarized in Figure 3. In the scanning electron microscopy images of the flour, used as the feed material, the starch granules appear as large spherical or kidney-shaped particles. Meanwhile, the protein bodies are present as small, mostly spherical particles, either individually dispersed or present in clusters. The particle size distribution of the flour (Figure 3) reveals a broad size distribution spanning from a few μm all the way to 100 μm. Two main populations of particles are present: the first ranging from 3 to 10 μm, and a second one from 10 to 40 μm. These can be attributed to non-starch components and to starch granules, respectively (2).

**Figure 2:**
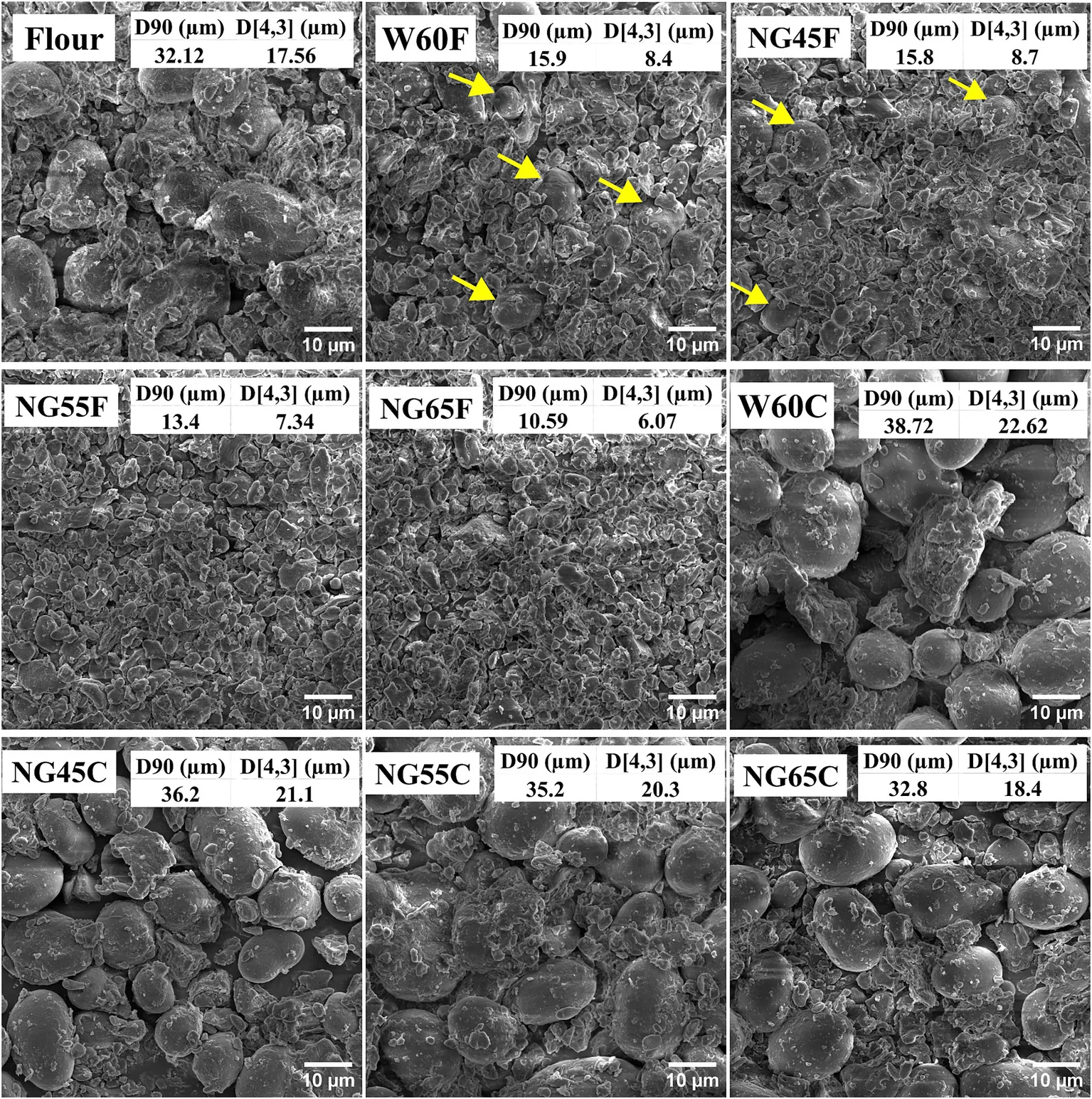
Scanning electron microscopy images of chickpea flour, fine (W60F, NG45F, NG55F, NG65F) and coarse (W60C, NG45C, NG55C, NG65C) fractions obtained during the four dry fractionation trials. D90 and volume-weighted mean diameter D[4,3] are reported for each sample. Yellow arrows highlight starch residues in the fine fractions.

**Figure 3:**
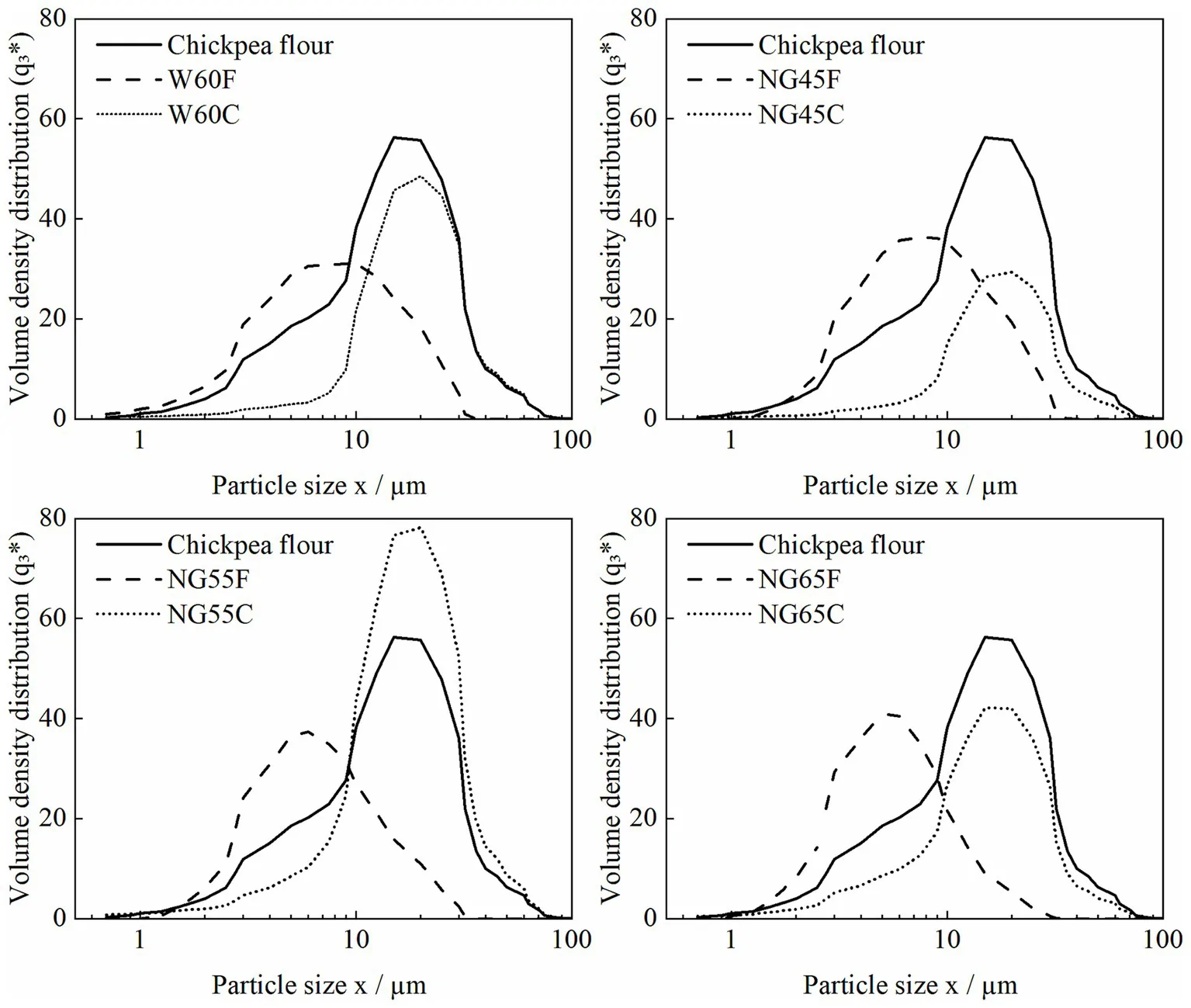
Volume density distribution of chickpea flour, fine (W60F, NG45F, NG55F, NG65F) and coarse (W60C, NG45C, NG55C, NG65C) fractions obtained during the four dry fractionation trials.

After the classification, all fractions showed a narrower size distribution compared to the initial flour. There was a clear distinction between the fine and coarse fractions, both microstructurally and in size distribution (Figures 2 and 3). In all cases, the protein-rich fraction showed much finer particles than the coarse fraction (Figure 3). Moreover, the particle size in the fine fractions NG55F and NG65F was reduced compared to W60F and NG45F. This was due to the higher concentration of small protein bodies obtained at higher classifier speed, confirming the compositional data. The D[4,3] and D_90_ of the coarse fractions were also reduced when moving from the wide to the NG wheel and increasing the NG wheel’s speed. This was because more protein bodies were discharged into the coarse fraction, when increasing the separation selectivity. Furthermore, a sharper profile of the fine fraction curve was visible at higher rotational speeds, with little difference between 5,500 and 6,500 rpm, confirming a more homogeneous particle size, given by the higher selectivity toward protein bodies.

Although the two initial particle populations were partially separated after the dry fractionation process, as observed in Figure 3, the fine fractions still showed a substantial degree of overlap with the coarse fractions. This overlap can be attributed to two factors: the presence of smaller or fragmented starch granules in the fine fractions, which were clearly visible in the SEM images of W60F and NG45F (Figure 2, yellow arrows), and the presence of residual protein, fragmented starch granules, or fiber as smaller particles within the coarse fraction. In particular, W60C and NG45C showed the presence of protein agglomerates and other non-starch components, observed either as discrete particles or attached to starch granules. This agglomeration is likely promoted by the high lipid content of chickpea flour, which increases powder cohesiveness, reducing the separability of the particles in the classifier chamber (2).

These results, in agreement with previous studies (2, 8), confirm that the main limit in the dry fractionation process of chickpea protein is the very broad size distribution and the greater overlapping size, compared to other legumes, of starch granules and protein bodies, making it quite challenging to obtain a clean separation of the two fractions.

### 3.3 Pasting and gelling properties of the coarse fractions

The differences in the compositional profile of the coarse fractions, particularly the starch and protein contents, are expected to directly influence their functional properties and potential for valorisation as a food ingredient. Unlike other studies focusing on the protein-enriched fine fraction, in this work, the gelation/pasting behavior of the coarse fraction was assessed by subjecting it to hydrothermal treatment, using a Rapid Visco Analyzer (RVA) (Figure 4). The pasting properties are summarized in Table 4. Pasting properties were determined according to AACC Method 22.02.02 (22). Three parameters were considered. Peak viscosity is the maximum viscosity reached during heating and reflects the swelling capacity of the starch granules, the possible contribution of non-starch carbohydrates, and the heat-induced aggregation of proteins. Trough viscosity is the minimum viscosity recorded during the holding period at constant temperature and indicates the stability of the system under prolonged heating and shearing. Final viscosity, measured at the end of the cooling stage, reflects the re-association of solubilized starch chains and the resulting network formation. All coarse fractions were compared at a dry matter content of 10% (w/w). As shown in Figure 4, all samples showed a rapid increase in viscosity at around 70 °C, reaching a first maximum at the end of the heating ramp, and showing a second increase in viscosity during cooling (for statistical analysis see Table 4). Interestingly, all NG coarse fractions showed very similar pasting properties regardless of the composition. Chickpea flour exhibited the lowest viscosity throughout the entire hydrothermal treatment, due to the lower starch concentration. On the other hand, W60C displayed not only the highest viscosity during heating, but also the steepest increase during the cooling stage. Since the samples were compared at constant dry matter, the observed differences are primarily attributed to variations in starch content and the starch-to-protein ratio (22). The high viscosity of W60C can be attributed to the highest concentration of starch (47.6 ± 1.9 g/100 g dry matter). A higher starch content increases the number of granules available for swelling and gelatinization, resulting in a higher peak viscosity; it also determines greater shear during heating (thus higher breakdown), enhancing amylose release during heating and its retrogradation during cooling, resulting in a higher final viscosity (23). In NG samples and the flour-based one, beyond the lower starch concentration, protein and lipids can influence the pasting behavior. Proteins can compete with starch for water and physically constrain granule swelling, leading to reduced viscosity development, breakdown, and setback (17, 18). In addition, lipids may form amylose-lipid complexes, which limit amylose leaching and delay gelatinization, further contributing to lower viscosity and reduced retrogradation (24).

**Figure 4:**
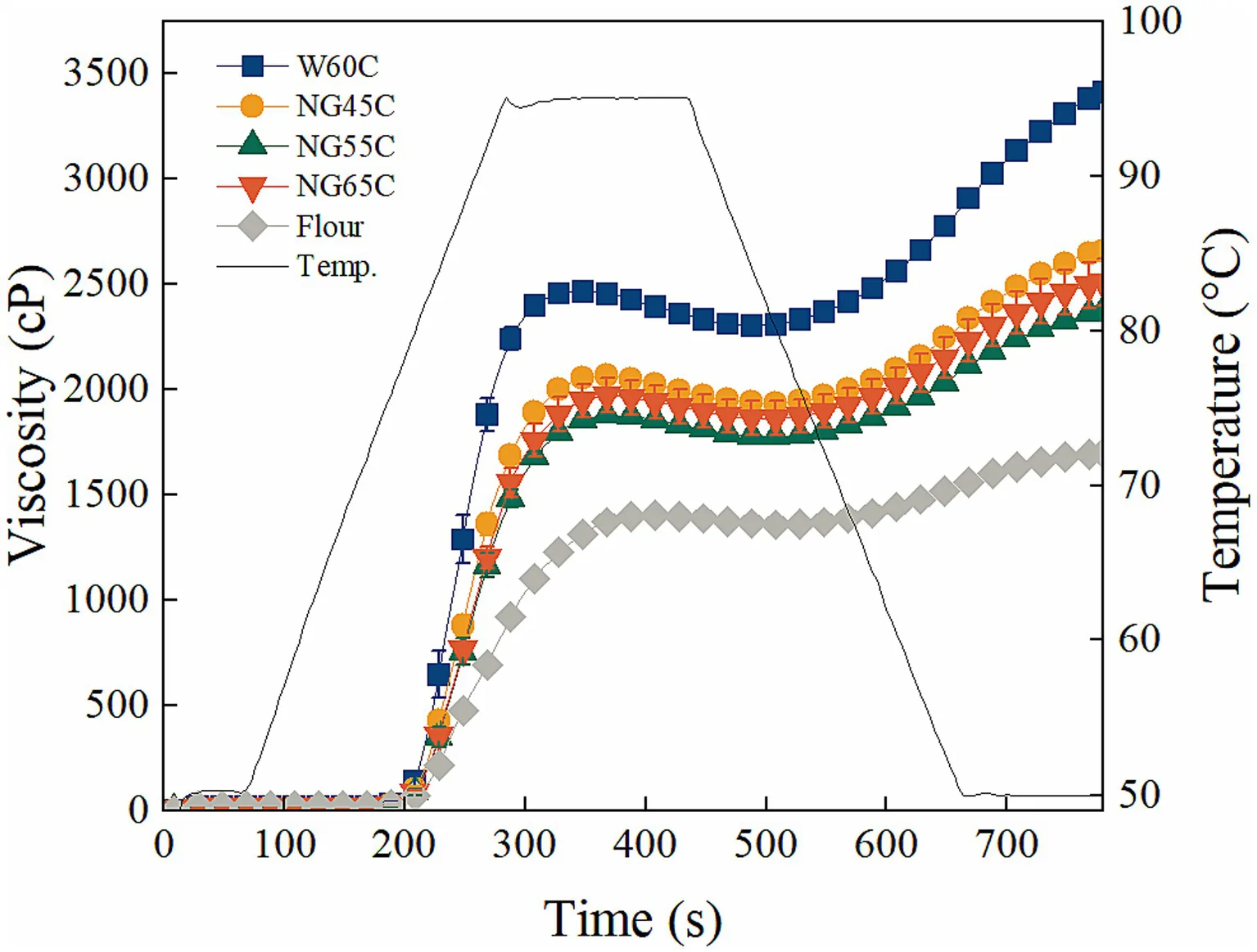
Pasting curves of chickpea flour and coarse fractions (W60C, NG45C, NG55C, NG65C) obtained during the four dry fractionation trials. Sample were obtained at 10% (w/w) on dry matter basis. Error bars indicate standard deviation.

**Table 4:** Parameters obtained during pasting properties carried out using a Rapid Visco Analyzer for the original chickpea flour and the four coarse fractions, tested at constant (10% w/w) dry matter.

Sample	PV (cP)	TV (cP)	FV (cP)
Flour	1,406 ± 6^d^	1,357 ± 8^d^	1702 ± 13^d^
W60C	2,470 ± 30^a^	2,304 ± 37^a^	3,413 ± 48^a^
NG45C	2067 ± 13^b^	1936 ± 11^b^	2,659 ± 34^b^
NG55C	1880 ± 3^c^	1776 ± 9^c^	2,373 ± 19^c^
NG65C	1915 ± 99^c^	1807 ± 97^c^	2,431 ± 135^c^

Peak viscosity (PV), trough viscosity (TV) and final viscosity (FV) are indicated. Within a column, different superscript letters indicate a significant difference (*p* < 0.05).

The results shown in Figure 4 highlight that the use of a wide classifier wheel not only provided the most favorable balance between fine fraction protein content, yield, and protein extraction efficiency, but also produced a coarse fraction with enhanced functional properties, attributable to its higher starch content. These results show the importance of considering the effect of functionality, and not only yields, in setting the processing parameters of the fractionation.

The microstructure of the gels obtained with 10% (w/w, dry matter basis) coarse fraction was analyzed by confocal laser scanning microscopy (CLSM) (Figure 5). All samples were characterized by a continuous matrix composed of swollen and gelatinized starch granules, within which protein bodies were embedded. The starch granules appeared elongated, which might be a consequence of the shear forces applied during the RVA analysis, while the protein-rich domains interrupting the matrix showed variable sizes and spatial distributions among the samples. The observed microstructure indicates that starch constituted the primary continuous phase in all samples, and proteins might be not fully hydrated, and still in their original aggregated state. Nevertheless, the presence of protein bodies within the starch matrix may locally disrupt matrix continuity and limit starch granule hydration through competition for water, contributing to structural heterogeneity within the gels. Although no major differences in overall matrix continuity were observed among samples, variations in the size and distribution of protein-rich domains suggest differences in local organization that may influence the structural properties of the gels.

**Figure 5:**
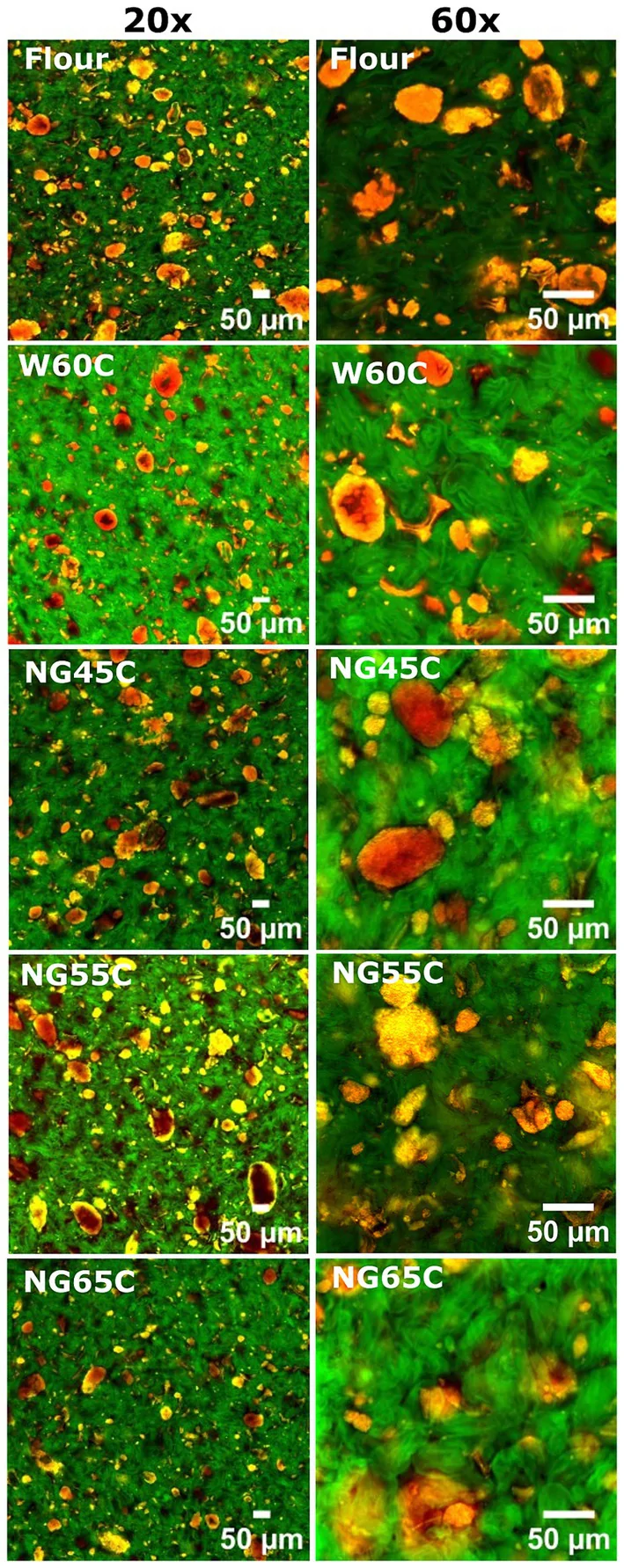
CLSM imaging of the gels obtained with chickpea flour and coarse fractions (W60C, NG45C, NG55C, NG65C). Samples were stained using fluorescein isothiocyanate (FITC, green) and Rhodamine B (red). FITC was used to highlight the starch-rich phase, whereas Rhodamine B to selectively stain protein. Images labeled “20×” (left column) were captured using a 20 × optical objective. Images labeled “60×” (right column) were obtained using 20 × optical objective combined with 3 × digital zoom (effective magnification equivalent to 60×).

In addition to the microstructure, the viscoelastic properties of the coarse fraction gels varied markedly across classifier configurations (Figure 6), showing a correspondence with their starch content, particularly for W60C. All samples exhibited a slightly frequency-dependent G’ and tan *δ* values below 0.15 across the entire tested frequency range, indicative of a predominantly elastic, stiff gel network (25). W60C displayed the highest G’ values, consistent with its elevated starch content relative to the other coarse fractions. Starch gelatinization is a major contributor to gel network formation in legume-based systems (26), while amylose retrogradation on cooling further reinforces the network (23). When the final gel viscoelastic properties were tested, the NG-coarse fractions showed similar values at the tested classifier rotational speed. The difference between all samples corresponded to what was observed with the pasting properties curves. Chickpea flour produced the weakest gel structure among the treatments, with the lowest G’ and the highest tan δ, suggesting that the non-starch components present in the unfractionated material, including proteins, dietary fiber, and lipids, interfere with starch network development. The frequency dependence of tan δ, particularly pronounced in the flour-based gel at high angular frequencies, further indicates a less stable network structure in these materials, likely attributable to its lower starch concentration. Taken together, these results showed that the main decrease in coarse fraction functionality (pasting viscosities and G′) occurred when moving from W60C to NG45C, with smaller changes at higher classifier speeds. Therefore, the further increase in fine fraction protein content at 5500 and 6,500 rpm (Table 3) was not associated with a substantial additional loss of functionality, but mainly with a lower fine fraction yield.

**Figure 6:**
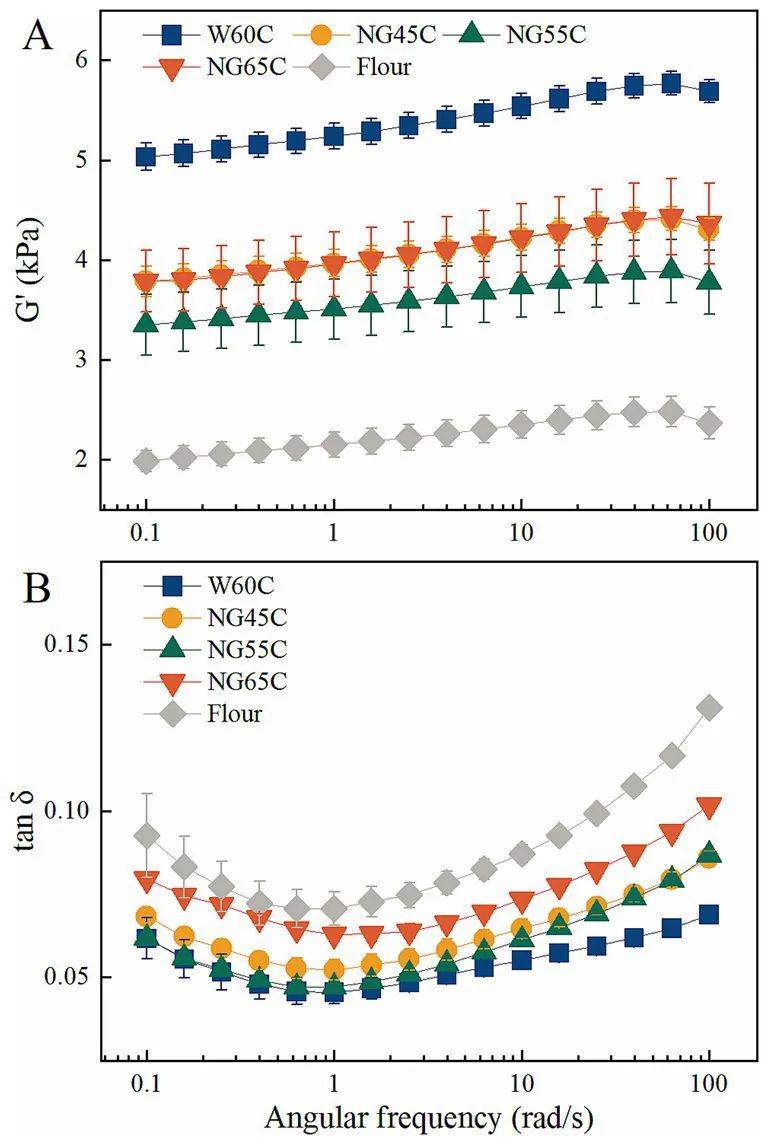
**(A)** Storage modulus (G’) and **(B)** tan δ values of the coarse fractions (W60C, NG45C, NG55C, NG65C) and flour gels, containing 10% (w/w) of dry matter. The gels were obtained after cooling the pastes obtained with Rapid Visco Analyzer hydrothermal treatment. Data shown are averages of three experiments with bars representing standard deviation.

### 3.4 Life cycle assessment

The results of the LCA analysis are presented in Table 5. The four different dry fractionation trials were first evaluated using two functional units: 1 kg of protein in the fine fraction and 1 kg of fine fraction. Based on the results, environmental impacts consistently increased from the least to the most refined fraction, across all studied midpoint categories, reflecting that the demand for higher protein enrichment directly impacts the environmental performance of the process.

**Table 5:** Results of the LCA analysis without allocation between the fine and coarse fractions. NG65F, NG55F and NG45F: fine fractions obtained with the new-generation classifier wheel at 6500, 5500 and 4500 rpm, respectively; W60F: fine fraction obtained with the wide classifier wheel at 6000 rpm. NG65C, NG55C and NG45C: coarse fractions obtained with the new-generation classifier wheel at 6500, 5500 and 4500 rpm, respectively; W60C: coarse fraction obtained with the wide classifier wheel at 6000 rpm.

Trial		W60F	NG45F	NG55F	NG65F	W60F	NG45F	NG55F	NG65F
Functional unit		1 kg of protein in fine fraction				1 kg of fine fraction			
Impact category	Climate change, default excluding biogenic carbon [kg CO_2_ eq.]	2.110	2.920	3.720	7.960	0.706	1.030	1.510	3.320
	Fine particulate matter formation [kg PM2.5 eq.]	0.0089	0.0123	0.0157	0.0336	0.0030	0.0043	0.0064	0.014
	Fossil depletion [kg oil eq.]	0.796	1.100	1.400	3.000	0.266	0.387	0.570	1.250
	Human toxicity, non-cancer [kg 1,4-DB eq.]	0.163	0.225	0.288	0.614	0.0545	0.0793	0.1170	0.2560
	Metal depletion [kg Cu eq.]	0.0018	0.0025	0.0032	0.0069	0.0006	0.0009	0.0013	0.0029
	Photochemical ozone formation, human health [kg NO_x_ eq.]	0.710	0.977	1.250	2.660	0.2370	0.345	0.508	1.110

When evaluating 1 kg of protein in the fine fraction, climate change impact increased by approximately 277%, rising from 2.11 and 2.92 kg CO₂ eq. for the fractions (W60F, and NG45F, respectively) of comparable refinement level, to 3.72 for NG55F, and to 7.96 kg CO₂ eq. for the fraction with the highest level of refinement (NG65F). Across this same range of trials, fine particulate matter formation grew by about 277% (0.0089 to 0.0336 kg PM2.5 eq.), fossil depletion rose from 0.796 to 3 kg oil eq., non-cancer human toxicity increased from 0.163 to 0.614 kg 1,4-DB eq., metal depletion grew from 0.0018 to 0.0069 kg Cu eq., and photochemical ozone formation increased from 0.710 to 2.660 kg NOₓ eq. These figures quantify how higher protein purity comes at the expense of a larger environmental footprint per unit of protein recovered.

Evaluating 1 kg of fine fraction revealed the same upward trend, though with a steeper relative increase. Climate change impact jumped by roughly 370%, moving from 0.706 and 1.03 kg CO₂ eq. for the least refined W60F and NG45F, to 1.510 for NG55F, and 3.320 kg CO₂ eq. for the highest extent of refinement (NG65F). Similarly, fine particulate matter increased from 0.0030 to 0.014 kg PM2.5 eq., fossil depletion rose from 0.266 to 1.250 kg oil eq., non-cancer human toxicity went from 0.0545 to 0.2560 kg 1,4-DB eq., metal depletion rose from 0.0006 to 0.0029 kg Cu eq., and photochemical ozone formation rose from 0.237 to 1.110 kg NOₓ eq.

The comparison between the two functional units highlights an important trade-off between product concentration and process efficiency. Expressing impacts per kilogram of protein results in higher values than expressing impacts per kilogram of fine fraction because the fine fractions contain only 36.5–46.1% protein and therefore more than 1 kg of product is required to deliver 1 kg of protein. However, the environmental implications of increasing protein purity cannot be interpreted from concentration alone. Increasing classifier selectivity from W60F to NG65F increased protein concentration from 36.5 to 46.1 g/100 g dry matter, but simultaneously reduced the fine-fraction yield from 36.19 to 7.70% and protein extraction efficiency from approximately 59 to 16%. Thus, achieving higher protein concentration resulted in substantially less efficient recovery of the protein present in the original feedstock. The additional processing intensity required to obtain a more concentrated fine fraction therefore needs to be considered against the associated loss of protein to the coarse fraction and the increased environmental burden per unit of product.

The two functional units provide complementary perspectives on the process. Measuring impacts per kg of fine fraction directly assesses the environmental burden of producing the physical output stream, which explains the sharper 370% climate change increase. Conversely, measuring per kg of protein, as higher protein extraction efficiency helps offset some of the added processing overhead, leading to a smaller 277% climate change increase. Evaluating both metrics highlighted the clear trade-off between processing intensity and environmental performance, while emphasizing the crucial role that protein concentration and yield play in calculating true sustainability.

While the scenarios above allocated the footprint to what was considered the main product of the fractionation, Table 6 summarizes the LCA values derived from the realistic scenario where the burden is allocated to both fine and coarse fractions, as both co-products of the fractionation. The LCA exhibited then a different pattern compared to the results above. Indeed, one needs to consider the coarse fraction to also be a valuable stream that can potentially be valorised as a co-product. In this case, the results were evaluated using the same two functional units, namely 1 kg of protein in the fine fraction and 1 kg of fine fraction.

**Table 6:** Results of the LCA analysis with mass allocation between the fine and coarse fractions. NG65F, NG55F and NG45F: fine fractions obtained with the new-generation classifier wheel at 6500, 5500 and 4500 rpm, respectively; W60F: fine fraction obtained with the wide classifier wheel at 6000 rpm. NG65C, NG55C and NG45C: coarse fractions obtained with the new-generation classifier wheel at 6500, 5500 and 4500 rpm, respectively; W60C: coarse fraction obtained with the wide classifier wheel at 6000 rpm.

Trial		W60F	NG45F	NG55F	NG65F	W60F	NG45F	NG55F	NG65F
Functional unit		1 kg of protein in fine fraction				1 kg of fine fraction			
Impact category	Climate change, default excluding biogenic carbon [kg CO_2_ eq.]	0.765	0.726	0.629	0.613	0.256	0.256	0.255	0.256
	Fine particulate matter formation [kg PM2.5 eq.]	0.0032	0.0031	0.0027	0.0026	0.0011	0.0011	0.0011	0.0011
	Fossil depletion [kg oil eq.]	0.288	0.273	0.237	0.231	0.0963	0.0962	0.0963	0.0963
	Human toxicity, non-cancer [kg 1,4-DB eq.]	0.0591	0.0559	0.0487	0.0473	0.0197	0.0197	0.0198	0.0197
	Metal depletion [kg Cu eq.]	0.0007	0.0006	0.0005	0.0005	0.0002	0.0002	0.0002	0.0002
	Photochemical ozone formation, human health [kg NO_x_ eq.]	0.257	0.243	0.211	0.205	0.0858	0.0858	0.0859	0.0855

When the functional unit of 1 kg of protein in the fine fraction was applied, the application of mass allocation resulted in the opposite outcome compared to the scenario above: a progressive decrease in environmental impacts from the least refined products to the highest refined fractions. For climate change, the impact decreased from the highest, 0.726 and 0.765 kg CO₂ eq. per kg of protein for NG45F and W60F to 0.629 and 0.613 kg CO₂ eq. per kg of protein for the highest refined fractions (NG55F and NG65F), as in this case the burden is no longer only on the fine fraction. This corresponds to a reduction of approximately 20% between the processes in focus. Similar trends are observed for all other impact categories. Fine particulate matter formation decreases from 0.0032 to 0.0026 kg PM2.5 eq. kg^−1^ protein, fossil depletion from 0.288 to 0.231 kg oil eq. per kg of protein, human toxicity, non-cancer from 0.0591 to 0.0473 kg 1,4-DB eq. per kg of protein, metal depletion from approximately 0.0007 to 0.0005 kg Cu eq. per kg of protein, and photochemical ozone formation from 0.257 to 0.205 kg NOₓ eq. per kg of protein. Thus, although the fractionation process becomes progressively more intensive and results in higher protein purity in the fine fraction from W60F to NG65F, the environmental burden attributed to each kg of recovered protein decreases when the coarse fraction is included as a co-product in the allocation procedure.

The results expressed per kg of fine fraction show an even more pronounced effect of mass allocation. Climate change remained remarkably stable across the four trials, with values of 0.256, 0.256, 0.255 and 0.256 kg CO₂ eq. per kg of fine fraction for W60F, NG45F, NG55F and NG65F, respectively. Similar behavior is observed for the other impact categories. Fine particulate matter formation remains at approximately 0.0011 kg PM2.5 eq. per kg of fine fraction, fossil depletion ranges from 0.0962 to 0.0963 kg oil eq., human toxicity, non-cancer from 0.0197 to 0.0198 kg 1,4-DB eq., metal depletion remains approximately 0.0002 kg Cu eq. kg^−1^, and photochemical ozone formation ranges from 0.0855 to 0.0859 kg NOₓ eq. per kg of fine fraction. The relatively narrow range of values indicates that, after accounting for the coarse fraction through mass allocation, the environmental burden per unit mass of fine fraction is largely independent of the fractionation trial.

The results obtained using economic allocation are presented in Table 7 and highlight the influence of the relative economic value of the fine and coarse fractions on the distribution of environmental burdens. Compared with mass allocation, economic allocation assigns a larger share of the processing-related impacts to the fine fraction due to its higher economic value per unit mass. For the functional unit of 1 kg of fine fraction, climate change impacts increased from 0.414 kg CO₂ eq. for W60F to 0.467, 0.509 and 0.554 kg CO₂ eq. for NG45F, NG55F and NG65F, respectively. Similar increasing trends were observed for fine particulate matter formation, fossil depletion, human toxicity, metal depletion and photochemical ozone formation. This increase is mainly associated with the progressively lower yield of the fine fraction at higher classifier selectivity, which results in a greater processing burden being associated with each kilogram of recovered fine fraction.

**Table 7:** Results of the LCA analysis with economic allocation between the fine and coarse fractions. NG65F, NG55F and NG45F: fine fractions obtained with the new-generation classifier wheel at 6500, 5500 and 4500 rpm, respectively; W60F: fine fraction obtained with the wide classifier wheel at 6000 rpm. NG65C, NG55C and NG45C: coarse fractions obtained with the new-generation classifier wheel at 6500, 5500 and 4500 rpm, respectively; W60C: coarse fraction obtained with the wide classifier wheel at 6000 rpm.

Trial		W60F	NG45F	NG55F	NG65F	W60F	NG45F	NG55F	NG65F
Functional unit		1 kg of protein in fine fraction				1 kg of fine fraction			
Impact category	Climate change, default excluding biogenic carbon [kg CO_2_ eq.]	1.24	1.32	1.25	1.38	0.414	0.467	0.509	0.554
	Fine particulate matter formation [kg PM2.5 eq.]	0.0052	0.0056	0.0053	0.0056	0.0018	0.0020	0.0022	0.0023
	Fossil depletion [kg oil eq.]	0.467	0.497	0.473	0.501	0.156	0.175	0.192	0.209
	Human toxicity, non-cancer [kg 1,4-DB eq.]	0.0956	0.102	0.0971	0.103	0.0319	0.0359	0.0394	0.0428
	Metal depletion [kg Cu eq.]	0.0011	0.0011	0.0011	0.0012	0.0004	0.0004	0.0004	0.0005
	Photochemical ozone formation, human health [kg NO_x_ eq.]	0.416	0.443	0.422	0.445	0.139	0.156	0.171	0.185

When the functional unit was defined as 1 kg of protein in the fine fraction, the differences between the trials were considerably smaller. Climate change impacts ranged from 1.24 kg CO₂ eq. for W60F to 1.38 kg CO₂ eq. for NG65F, while the remaining impact categories followed similar patterns. These results demonstrate that higher protein enrichment does not necessarily translate into improved environmental performance, as the benefits of increased protein concentration must be considered together with the corresponding reduction in fine-fraction yield and protein extraction efficiency. Overall, the economic allocation results confirm that the environmental performance of dry fractionation is governed by the combined effects of product yield, protein concentration, recovery and relative economic value, rather than by protein enrichment alone. Therefore, the selection of the optimal classifier configuration should consider the trade-off between obtaining a protein-enriched fraction and minimizing the environmental burden associated with its production.

Overall, the results demonstrate that the treatment of the coarse fraction has a substantial influence on the environmental performance attributed to the protein-rich fine fraction. In the scenario without allocation, increasing fractionation intensity led to progressively higher environmental impacts because the entire burden of the process is assigned to the fine fraction. In contrast, when the coarse fraction is considered a valuable starch-rich co-product and mass allocation is applied, part of this burden was transferred to the coarse fraction. Consequently, the environmental impact per kg of fine fraction becomes almost constant across the different trials, while the impact per kg of protein decreases with increasing fractionation intensity. Moreover, under the economic allocation scenario, the environmental burden is distributed between the fine and coarse fractions according to their relative economic values rather than solely their mass shares. Consequently, the environmental impact per kg of fine fraction increases with increasing fractionation intensity, reflecting the progressive reduction in fine-fraction yield and the relatively higher economic value assigned to the protein-enriched fraction. Similarly, the impact per kg of protein remains relatively stable but shows an overall increase at higher fractionation intensities, indicating that the gains in protein concentration do not fully compensate for the lower product yield and the greater share of environmental burdens allocated to the fine fraction. These results suggest that evaluating the dry fractionation process while considering both valuable output streams provides a more representative assessment of its environmental performance and highlights the potential environmental benefits associated with valorising the coarse fraction as a co-product.

## 4 Conclusion

This study evaluated four classifier configurations for dry fractionation of chickpea flour, integrating process performance, coarse fraction functionality, and environmental impact. The results demonstrated a clear trade-off between fine fraction refinement level, coarse fraction functionality, and the environmental impact of the entire process. The results show that optimizing dry fractionation solely for protein concentration is not always the most effective strategy, as it may reduce coarse fraction functionality and increase the environmental burden of the dry fractionation process. In fact, the coarse fractions exhibited distinct functional properties depending on the applied process parameters. All coarse fractions demonstrated high potential for use as food-structuring agents. This highlights the importance of considering the coarse fraction as a valuable and functional output of the fractionation process, rather than merely as a side stream, and of incorporating its functionality into the overall optimization of dry fractionation processes. The LCA results further demonstrated the importance of considering both fractions as relevant process outputs. When the environmental burden was attributed entirely to the fine fraction, increasing fractionation intensity resulted in higher environmental impacts. However, when the coarse fraction was instead treated as a co-product, this trend changed. Under mass allocation, the impact per kg of protein decreased as refining increased, while under economic allocation, which accounts for the higher value of the protein-rich fraction, it showed a minimal increment. How the coarse fraction is valorised therefore determines how the different configurations compare in environmental terms. Future research should move beyond evaluating dry fractionation solely on fine fraction yield and functionality, and instead tailor process conditions to the functional requirements of specific food applications, rather than maximizing protein enrichment. By defining the intended use of both fine and coarse fractions beforehand, process parameters could be optimized to achieve only the degree of refinement required for the target formulation, potentially reducing unnecessary processing intensity. Furthermore, integrating the intended applications of the resulting fractions into the LCA could provide a more representative assessment of their environmental performance and valorisation potential. This study represents a first step in this direction, since the trials were performed with industrial scale equipment operated in batch mode. Coupling this approach with trials in a continuous line would allow a quantitative techno-economic analysis, enabling a more comprehensive evaluation of the economic and environmental trade-offs associated with different fractionation strategies.

## Data Availability

The datasets presented in this study can be found in online repositories. The names of the repository/repositories and accession number(s) can be found at: 10.5281/zenodo.22200674.
